# Advice about diet and smoking for people with or at risk of age-related macular degeneration: a cross-sectional survey of eye care professionals in the UK

**DOI:** 10.1186/1471-2458-13-564

**Published:** 2013-06-10

**Authors:** John G Lawrenson, Jennifer R Evans

**Affiliations:** 1 Division of Optometry and Visual Science, City University London, Northampton Square, London, UK; 2 International Centre for Eye Health, London School of Hygiene and Tropical Medicine, Keppel St, London, UK

**Keywords:** Age-related macular degeneration, Lifestyle modification, Nutrition, Smoking cessation

## Abstract

**Background:**

In the absence of a cure, there has been considerable interest in attempts to prevent or reduce the progression of age-related macular degeneration (AMD) by targeting particular modifiable risk factors. The aim of this study was to conduct a cross-sectional survey of the current practice of UK eye care professionals in relation to advice given on diet and other lifestyle modifications for patients with or at risk of AMD.

**Methods:**

Optometrists and ophthalmologists on the membership databases of professional organisations for the two professions were invited to participate in an online survey. The survey was open for 12 weeks between July and September 2012.

**Results:**

A total of 1,468 responses were received (96.3% from optometrists and 3.7% from ophthalmologists). The response rate of those receiving the invitation was 16.2% (1,414/8735) for optometrists and 6% (54/1460) for ophthalmologists. A majority of respondents reported that they frequently provide dietary advice to patients with established AMD (67.9%) and those at risk of AMD (53.6%). Typical advice consisted of a recommendation to eat plenty of leafy green vegetables and eat more oily fish. The decision to recommend nutritional supplements was based on the risk of progression to advanced AMD, with approximately 93% of respondents recommending supplementation in a patient with advanced AMD in one eye. However for the majority, the type of supplement recommended did not comply with current best research evidence, based on the findings of the Age-related Eye Disease Study (AREDS). Only one in three optometrists regularly assessed smoking status and advised on smoking cessation.

**Conclusions:**

Within a large sample of eye care professionals, consisting predominantly of optometrists, who responded to a cross-sectional survey, there was active engagement in providing nutritional advice to patients with or at risk of AMD. However, the results demonstrate a need to raise awareness of the evidence underpinning the use of nutritional supplements together with an increased involvement in targeted smoking cessation.

## Background

Age-related macular degeneration (AMD) is the leading cause of visual impairment in Western countries and with an ageing population the numbers of people affected are projected to rise [1]. Although in recent years, effective therapeutic interventions have been developed that can stabilize vision in those with the neovascular (‘wet’) form of the disease, for the majority of people affected no treatment is currently available [2]. In its advanced form, AMD has significant impact on quality of life and profoundly limits an individual’s ability to function independently [3]. As a consequence, there has been a great deal of interest in the identification and targeting of modifiable risk factors for AMD for both disease prevention and to reduce the risk of progression to advanced AMD [4].

Epidemiological studies have identified a variety of lifestyle exposures that have been putatively linked to the development and progression of AMD, including smoking [5,6] and nutritional factors [7]. Smoking has been identified as the most consistently reported modifiable risk factor for the development of AMD and increasing the risk of progression to advanced AMD. By contrast, the role of diet and nutritional supplementation is much less clear. Data from observational studies provide inconsistent evidence for a protective role of dietary antioxidants [8-10] and results from randomised controlled trials of nutritional interventions are less encouraging. There is currently no evidence from randomised intervention studies to support the use of nutritional supplements in primary prevention or to slow progression in patients with early AMD [11,12], however for populations at a higher risk of progression to advanced disease, the use of high dose antioxidant vitamin and zinc supplementation has been shown to be protective [13,14].

Dietary supplements are widely marketed as a strategy for AMD prevention and treatment and very little reliable information is available to guide the public in making the decision as to whether or not to take these supplements. Eye care practitioners are often in a position where they have to provide information and advice to patients with diagnosed AMD or at risk of developing the disease, on the benefit of specific nutritional interventions or other lifestyle changes. However, the quality of the evidence supporting these measures is variable and often contradictory, which could lead to a lack of consistency in the advice given. The primary aim of the present study was to conduct a cross-sectional survey of UK optometrists and ophthalmologists to investigate current practice in relation to the targeting of modifiable risk factors in AMD. A secondary aim of the study was to identify the sources of evidence that practitioners use to inform their recommendations in this area.

## Methods

The survey was delivered entirely online and was hosted by Survey Monkey (a US provider of web-based surveys http://www.surveymonkey.com). An initial version of the survey was developed by the authors and piloted prior to issuing the final version An invitation to take part in the survey was included as a news item in an electronic newsletter that is sent periodically to members of the College of Optometrists and an email news bulletin sent to UK consultant ophthalmologists by the Royal College of Ophthalmologists. Both newsletters included a hyperlink to the survey home page. An incentive in the form of a prize draw for £100 in shopping vouchers was offered to those completing the survey. Ethical approval for the study was granted by the City University London School of Health Sciences Research and Ethics Committee and the research was carried out in compliance with the Declaration of Helsinki (http://www.wma.net/en/30publications/10policies/b3/index.html).

The survey consisted of 18 forced choice questions and one free text question divided into 4 sections:

1. Dietary advice (4 questions): questions in this section asked if practitioners offered dietary advice to their patients with diagnosed AMD or those at risk of AMD, follow-up questions asked about specific advice from a forced-choice list. The list included advice to increase consumption of leafy green vegetables (a rich source of the macular carotenoid lutein), increased intake of oily fish (source of omega 3 fatty acids) or both of the above. A free-text option was provided to specify any additional dietary advice given.

2. Recommendations on the use of nutritional supplements (6 questions): these questions were based on the presentation of 3 case scenarios presenting patients with varying risk of progressing to advanced AMD:

a. 55-year old patient with no evidence of AMD but with one or more parents and/or siblings affected by AMD

a. 65-year old patient with advanced AMD in one eye and early AMD in the other

a. 75-year old patient with advanced AMD in both eyes

Respondents were firstly asked if they would advise nutritional supplements for each specified patient and if so, which supplement they would recommend from a list provided. The scenarios were chosen to reflect the available evidence-base on the value of nutritional supplementation in AMD.

The specified list of supplements included those most widely available in the UK:

AREDS formula e.g. *Bausch & Lomb Preservision*

Supplement containing macular carotenoids lutein and zeaxanthin e.g. *Macushield*

Supplement containing antioxidant vitamins, lutein and zeaxanthin e.g. *ICaps*

Supplement containing omega 3 fatty acids e.g. *Ocuvite Complete*

The Age-related Eye Disease Study (AREDS) formulation is the only supplement in the list for which evidence of effectiveness is available from randomised controlled trials (RCTs) [14]. The composition and doses of nutrients within each of the specified supplements are given in Table 1.

3. Smoking and AMD (5 questions): this section investigated practice in relation to taking a smoking history, whether practitioners explained to patients the relationship between smoking and AMD, and whether they provided advice on smoking cessation. The final question asked if practitioners took a patient’s smoking history into account when recommending nutritional supplements. There is good evidence that smokers who take beta-carotene may be at increased risk of developing lung cancer [15] and therefore it is recommended that current or past smokers should be advised to use a supplement that does not contain beta-carotene.

4. Evidence base for nutritional supplement interventions (4 questions): in this section, respondents were asked to rate the strength of evidence to support the use of nutritional supplements in the prevention or treatment of AMD. The terminology adopted by the GRADE working group [16] for quality of evidence (high, moderate, low, very low) was used (see Table 2 for an interpretation of the GRADE categories). The second part of this section sought to identify the specific sources of evidence used by respondents to inform their views regarding the benefits of supplementation.

**Table 1:** Composition of supplements specified in the survey

**Nutrient**^**§**^	B&L Preservision*	***Macushield***	***ICaps***	***Ocuvite Complete***
Vitamin C	452	-	125	180
Vitamin E	268	-	50	30
Vitamin A	17.2	-	0.8	-
Vitamin B2	-	-	1.4	-
Zinc	69.2	-	20	15
Selenium	-	-	0.05	-
Manganese	-	-	2	-
Lutein	-	10	10	10
Zeaxanthin	-	2	**	2
Meso-zeaxanthin	-	10	-	-
Omega 3 fatty acids	-	-	-	500

^§^ composition in mg, *Formulation equivalent to that used in AREDS., ** specified as Lutein/zeaxanthin 10mg.

**Table 2:** Details of the quality of evidence categories in GRADE

**GRADE**	**Descriptor**
High	Further research is very unlikely to change our confidence in the estimate of effect
Moderate	Research is likely to have an important impact on our confidence in the estimate of effect and may change the estimate
Low	Further research is very likely to have an important impact on our confidence in the estimate of effect and is likely to change the estimate
Very low	Any estimate of effect is very uncertain.

The survey was set up such that participants could not go back to change the answer to any question once it was submitted. Although respondents could exit the survey at any time, responses to previously answered questions were automatically saved.

The survey was open for 12 weeks between July-September 2012. An email reminder was sent approximately one week after the initial invitation.

The survey data was downloaded into an Excel spreadsheet and prepared for analysis. Responses to forced choice questions were analysed as simple proportions of all valid responses and where appropriate, according to whether the respondent was an optometrist or an ophthalmologist. Free text responses were also downloaded, coded and then assigned to categorical variables by the lead author. The text files obtained from the free-text responses from optometrists and ophthalmologists were also used to generate a visual representation (‘word cloud’). Word clouds give greater prominence to words that appear more frequently in the source text (http://www.wordle.net/).

Statistical analysis of differences between the responses of optometrists and ophthalmologists was carried out using a Chi-square test and applying Yates’s correction.

## Results

A total of 1,468 (full or partial) responses were received (1,414 (96.3%) from optometrists and 54 (3.7%) from ophthalmologists). The invitation to take part in the survey was delivered via an e-newsletter/electronic bulletin sent to 8,735 members of the College of Optometrists and 905 UK consultant ophthalmologists by the Royal College of Ophthalmologists. The response rate of those receiving the invitation was 16.2% for optometrists and 6% for ophthalmologists.

### Dietary advice

The majority (67.9%) of respondents reported that they would always (or usually) provide dietary advice to patients with established AMD, with over half (53.6%) regularly offering advice to those considered to be at risk of AMD (Table 3). The specific advice given to each group was similar; with most respondents advising patients to eat plenty of leafy green vegetables and oily fish at least twice a week. Free text responses most commonly referred to recommendations on eating coloured fruits and vegetables e.g. yellow peppers, bilberries and blueberries.

**Table 3:** Dietary advice to patients with established AMD or at risk of AMD

**Dietary advice**	**N (%)**
**Frequency of dietary advice for patients with established AMD**	
o Always/most of the time	917 (67.9)
o Sometimes	383 (28.4)
o Never	50 (3.7)
**Frequency of dietary advice for patients considered to be at risk of AMD**	
o Always/most of the time	719 (53.6)
o Sometimes	526 (39.2)
o Never	97 (7.2)
**Dietary advice for patients with established AMD**	
o Eat plenty of leafy green vegetables	497 (38.3)
o Eat oily fish at least twice per week	3 (0.2)
o Both of the above	652 (50.7)
o Other	140 (10.8)
**Dietary advice for patients considered to be at risk of AMD**	
o Eat plenty of leafy green vegetables	476 (38.4)
o Eat oily fish at least twice per week	6 (0.5)
o Both of the above	585 (47.2)
o Other	173 (14.0)

No differences were found between responses for optometrists and ophthalmologists in relation to dietary advice given.

### Recommendations on the use of nutritional supplements

The % of respondents recommending nutritional supplements for different types of patient is given in Table 4 and the frequency of particular supplement recommendations is provided in Table 5. The results suggest that the likelihood of a patient being advised to take a supplement is dependent on their risk of progression to advanced AMD; approximately 34% of respondents reported that they recommended supplementation for primary prevention in a patient with a family history of AMD, compared to approximately 93% for a patient with advanced AMD in one eye and early AMD in the other. The most commonly reported supplement for people with or at risk of AMD was either a preparation containing macular carotenoids only or one containing lutein and zeaxanthin in combination with antioxidant vitamins. The supplement used in the AREDS trial (AREDS formula) was one of the least likely to be recommended (Table 5). For example, in those respondents recommending a single supplement, the AREDS formula was recommended by only 13.5% in the scenario describing a patient with advanced AMD in one eye.

**Table 4:** Recommendations on nutritional supplements in patients with established AMD or at risk of developing AMD

**Patient scenarios**	**% of respondents recommending supplements**
55-year old patient with no evidence of AMD but with one or more parents and/or siblings affected by AMD	33.6
65-year old patient with advanced AMD in one eye and early AMD in the other	92.8
75-year old patient with advanced AMD in both eyes	44.8

**Table 5:** Specific supplement recommendations for each of the 3 patient scenarios

**Supplement recommendations**	**N (%)**
**55-year old patient with no evidence of AMD but with one or more parents and/or siblings affected by AMD**	
o AREDS formula	72 (15.6)
o Supplement containing macular carotenoids	287 (62.1)
o Supplement containing antioxidant vitamins, lutein and zeaxanthin	291 (63.0)
o Supplement containing omega 3 fatty acids	93 (20.1)
**65-year old patient with advanced AMD in one eye and early AMD in the other**	
o AREDS formula	348 (27.5)
o Supplement containing macular carotenoids	818 (64.6)
o Supplement containing antioxidant vitamins, lutein and zeaxanthin	724 (57.2)
o Supplement containing omega 3 fatty acids	262 (20.7)
**75-year old patient with advanced AMD in both eyes**	
o AREDS formula	161 (26.8)
o Supplement containing macular carotenoids	395 (65.8)
o Supplement containing antioxidant vitamins, lutein and zeaxanthin	340 (56.7)
o Supplement containing omega 3 fatty acids	150 (25.0)

**Figure 1:**
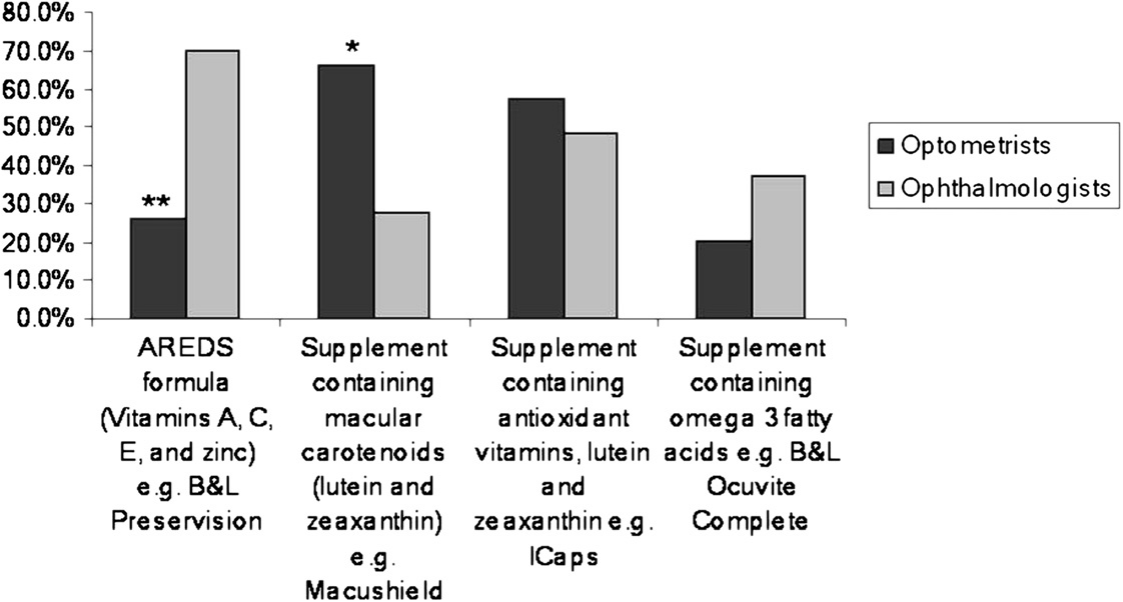
Frequency of particular supplement recommendations for a patient with advanced AMD in one eye and early AMD in the other (** p=0.0001. *p=0.0072).

Although 96.3% of survey respondents were optometrists, a sub-analysis comparing responses from optometrists and ophthalmologists revealed that the latter were significantly less likely to recommend supplementation for primary prevention (only 9.6% of respondents recommending a supplement for a patient at risk of developing AMD by virtue of family history compared to 34.6% of optometrists (difference significant p=0.0061)). However when a supplement was recommended, there was a greater likelihood that ophthalmologists would recommend the AREDS formula (Figure 1).

### Smoking and AMD

Overall, 32.3% of respondents reported regularly taking a smoking history in new patients and 21.2% in review patients. However, a greater proportion (49.4%) indicated that they frequently informed smokers of the link between smoking and eye disease (Table 6). By contrast, only a third of respondents regularly advised smokers to quit. The final question in this section asked if smoking history was taken into account when recommending nutritional supplements. 70.3% responded positively to this question.

**Table 6:** Results of responses relating to smoking and eye disease

**Smoking advice**	**N (%)**
**Frequency of taking a smoking history in new patients**	
o Every/most of the time	431 (32.3)
o Sometimes	533 (40.0)
o Rarely/never	369 (27.7)
**Frequency of taking a smoking history in review patients**	
o Every/most of the time	283 (21.2)
o Sometimes	576 (43.2)
o Rarely/never	474 (35.6)
**Frequency of informing smokers of the link between smoking and eye disease**	
o Every/most of the time	658 (49.4)
o Sometimes	546 (41.0)
o Rarely/never	129 (9.7)
**Frequency of advising patients to stop smoking**	
o Every/most of the time	449 (33.7)
o Sometimes	539 (40.8)
o Rarely/never	345 (25.9)

**Figure 2:**
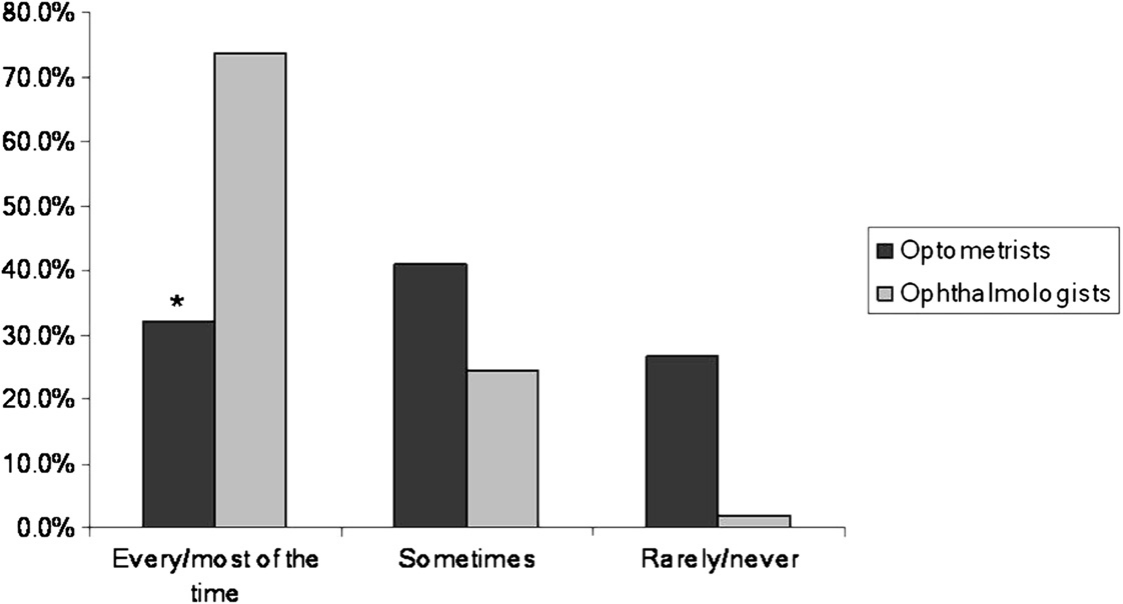
Frequency of taking a smoking history in new patients. (*p=0.0002).

A sub-analysis of differences between optometrists and ophthalmologists showed that ophthalmologists were significantly more likely to take a smoking history from their patients (Figure 2). Furthermore, 70-80% of ophthalmology respondents frequently explained the relationship between smoking and eye disease and advised their patients to quit.

### Evidence base for nutritional supplement interventions

The four questions in this section concerned the evidence base for the use of nutritional supplements in AMD and the sources of evidence used to inform practice (Tables 7 and 8). The majority of respondents rated the quality of evidence supporting the use of nutritional supplements for both prevention and slowing the progression of AMD as moderate. Articles in professional journals and conference presentations were the most frequently cited sources that have informed practitioner views on the role of nutritional supplements in AMD. Free text responses also commonly referred to professional journals as the primary evidence source with the majority of optometrists specifically mentioning non-peer reviewed professional magazines e.g. *Optometry Today* and *Optician*. A significant proportion (15.1%) also cited the use of manufacturer’s product literature as an evidence source. There were numerous references to specific supplementation studies, in particular AREDS (21.3%). Other sources of evidence less frequently reported included: personal experience, university undergraduate/postgraduate lectures, talking to colleagues and literature provided by AMD support groups.

**Table 7:** Results of responses rating the quality of evidence supporting the use of nutritional supplements in AMD

**Quality of evidence**	**N (%)**
**Rating of the strength of evidence supporting the use of supplements for AMD prevention**	
o High quality	185 (13.9)
o Moderate quality	873 (65.6)
o Low quality	247 (18.6)
o Very low quality	25 (1.9)
**Rating of the strength of evidence supporting the use of supplements for slowing the progression of AMD**	
o High quality	215 (16.2)
o Moderate quality	849 (64.1)
o Low quality	239 (18.0)
o Very low quality	22 (1.7)

**Table 8:** Sources of evidence informing practitioner views on nutritional supplements and AMD

**Sources of evidence**	**(%)**
**Free text response**	n = 1,196
o Articles in professional journals	40.2
o Conference presentations, CE events	25.9
o Reference to specific studies e.g. AREDS	21.3
o Scientific/Research literature	16.4
o Manufacturers Literature	15.0
o Expert opinion	11.7
o Cochrane Reviews	0.8
o Medline	0.8
o NICE guidance	0.3
o Other	18.6
**Responses to forced choice question**	n=1,245
o Articles in professional journals	88.4
o Expert opinion	46.1
o Conference presentations	62.9
o NICE guidance	23.1
o Medline	7.7
o Cochrane Reviews	5.7
o None of the above	2.3

Abbreviations: *CE*=Continuing education, *NICE*= National Institute for Health and Clinical Excellence, *AREDS*=Age-related Eye Disease Study.

**Figure 3:**
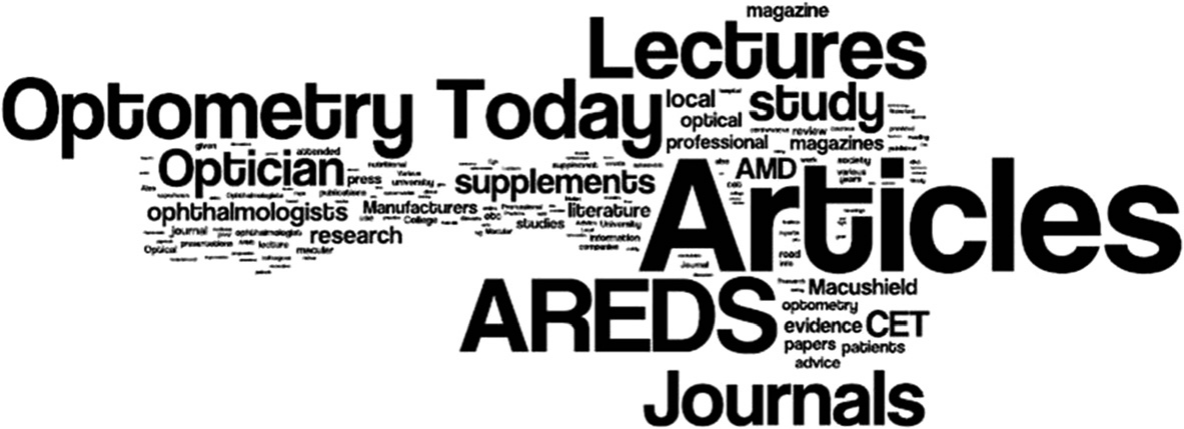
Word cloud generated from free-text responses from optometrists (N=1,196) to a question asking about sources of evidence used to inform recommendations on the use of nutritional supplements.

**Figure 4:**
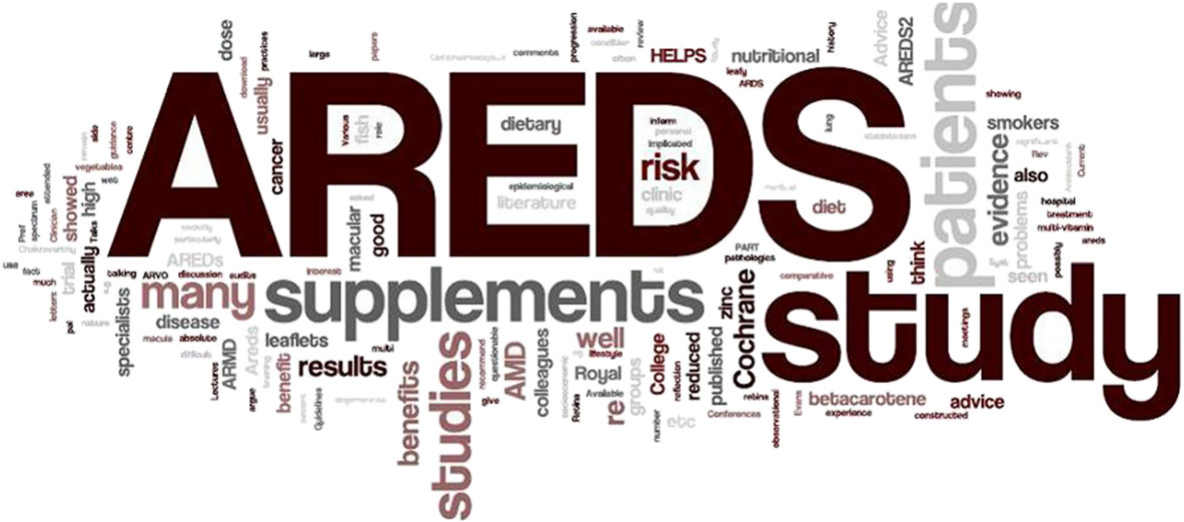
Word cloud generated from free-text responses from ophthalmologists (N=47) to a question asking about sources of evidence used to inform recommendations on the use of nutritional supplements.

A visual representation of the free-text responses of optometrists and ophthalmologists is shown in Figures 3 and 4.

## Discussion

The current study is the first to systematically investigate the extent to which UK eye care practitioners provide advice on modifiable risk factors for AMD. The survey questions were grouped into sections, covering advice on nutrition (including recommendations regarding nutritional supplements), attitudes and behaviours of optometrists and ophthalmologists towards smoking cessation and an exploration of the use of evidence to inform clinical decision making.

The invitation to participate in the survey was delivered via electronic newsletters issued by UK professional bodies for optometry and ophthalmology. Although the membership database of these organisations reflects the demographics of these professions, since the survey was anonymous and personal details of respondents were not collected, it was not possible to establish if the sample of people who responded to this survey was representative of everyone on the membership database. Furthermore, the low response rate from ophthalmologists means that the survey findings predominantly reflect the current practice of optometrists. The response rate from optometrists is consistent with previous studies, which have reported response rates in the range 12-28%. Furthermore, the demographics of those responding to these surveys have been consistent with the national register of optometrists in terms of age and gender and geographic location [17-19]. However, it is possible that those who responded to the present survey had a particular interest in AMD. It is difficult to be sure what effect this would have on the results but it is most likely that the frequency of reporting advice on nutrition and lifestyle is overestimated rather than underestimated in this survey. However, we think it unlikely that the information on type of supplement recommended would be affected by response bias.

Approximately two thirds of respondents reported that they frequently offered dietary advice to those with established AMD and over half in those considered to be at risk. Advice given most often consisted of a recommendation to consume plenty of leafy green vegetables. The rationale behind dietary modification in AMD is principally to increase the intake of antioxidant nutrients and more specifically to raise levels of the macular carotenoids, lutein and zeaxanthin. Lutein and zeaxanthin are the principal components of macular pigment which plays an important role in visual function and also possesses essential antioxidant and protective blue-light filtering properties [20]. These macular carotenoids are not synthesised by the body *de novo* and therefore humans rely solely on dietary intake. The richest sources of lutein and zeaxanthin are from leafy green vegetables, such as spinach and kale, and orange or yellow fruits and vegetables. Randomised intervention trials investigating the impact of diet on AMD are uncommon and often target single nutrients. Consequently, the evidence for the role of diet in AMD is based largely on observational studies and is therefore subject to confounding and bias. However, until better quality evidence becomes available, it appears that UK eye care practitioners have taken the view that a recommendation to eat more leafy green vegetables and increase omega 3 fatty acid intake is beneficial, with minimal possibility of harm. This view is also consistent with current professional guidance [21,22].

For certain individuals, it may be difficult to obtain adequate levels of antioxidants and other essential nutrients through diet alone. In such cases, there could be some advantage in augmenting particular nutrients through supplementation. Evidence supporting the use of supplementation in AMD comes primarily from the AREDS trial [13], which demonstrated that a supplement containing high doses of vitamin C, vitamin E, beta-carotene and zinc could reduce progression to advanced AMD by 25% in populations with intermediate AMD or advanced AMD in one eye. The present survey presented three scenarios of patients with varying degrees of risk of progression to advanced AMD along with a question asking whether they would recommend a supplement and if so, which one from a supplied list. The results demonstrated that the decision to recommend supplementation was based on the likelihood of disease progression, with approximately 93% of respondents reporting that they would recommend a supplement in a person with advanced AMD in one eye and early AMD in the other. However, despite the availability of evidence from the AREDS trial that this category of patient would benefit from a formulation containing high dose vitamins and zinc (AREDS formula), this particular supplement was one of the least likely to be recommended. Instead, the majority of respondents reported that they advised patients to take a supplement containing macular carotenoids only or a product containing lutein and zeaxanthin in combination with antioxidant vitamins. However, the latter typically contain lower doses of antioxidant vitamins than found in the AREDS formula.

Approximately 96% of survey responses were received from optometrists and therefore the pooled data primarily reflects the practice of optometrists. However, a sub-analysis of ophthalmologist responses showed significant differences in the type of supplement recommended. Specifically, for the scenario describing a patient with advanced AMD in one eye and early AMD in the other, the AREDS formula was the most likely to be recommended by ophthalmologists (approximately 70% of selections), compared to only 26% by optometrists. Although it is possible that this discrepancy is due to response bias, the magnitude of the difference suggests a different approach to supplementation by the two professions. A possible explanation is that many optometrists may be unaware of the evidence-base supporting the use of the AREDS formula. However, from the optometrist’s free-text responses to the question asking about evidence sources used to inform decision making, it was clear that there was a high level of awareness of the AREDS trial, which could explain the large number of respondents who recommended supplementation in a patient at high risk of AMD progression. However, this awareness did not translate into recommending an AREDS-type supplement. Another possible explanation is that optometrists may have chosen an alternative supplement due to the potential for adverse reactions associated with the AREDS formula. There have been well-publicised concerns regarding the use of supplements containing doses of vitamins and minerals that are significantly higher than the recommended daily allowance (RDA).

It is important that any advice given to patients regarding lifestyle modifications and particularly recommendations on the benefits of nutritional supplementation is informed by the best available research evidence. The most commonly cited sources of evidence in the survey were lectures or conference presentations and articles in professional journals. Large numbers of optometrist respondents referred specifically to professional ‘magazines’ that are produced either weekly or fortnightly and contain non peer-reviewed clinical articles. It may also be of significance that these periodicals frequently contain featured articles sponsored by manufacturers and advertisements for a variety of alternative nutritional products.

Most research concerning the evidence-based practice and information-seeking behaviour of healthcare professionals has focussed on nurses and doctors. However, a systematic review examining factors which determine the application of research evidence by allied health professionals, identified level of academic qualification, involvement in research and practitioners attitudes and beliefs about evidence based practice as significant predictors of the use of research evidence in practice [23]. With regard to the specific use of evidence by optometrists, although a recent. Australian study found generally positive attitudes towards evidence-based practice, most practitioners reported a reliance on information gained during undergraduate or postgraduate training or continuing education courses to inform their clinical decision-making [24].

In addition to the well documented harmful effects of smoking on the cardiovascular and respiratory systems, there is increasing evidence that smoking is causally linked to the development of AMD [25]. Smoking increases the risk of AMD two-fold and based on the UK population, it has been estimated that approximately 28,000 cases of AMD in older people may be directly attributable to smoking [26]. Since public awareness of the link between smoking and ocular health is lacking [27-29], eye care professionals play a critical role in educating the public and encouraging smokers to quit. However, only a third of respondents in the current survey reported that they regularly took a smoking history in new patients and a similar number were proactive in advising on smoking cessation. Previous studies that have investigated the attitudes and practice of optometrists in this area have reported similar findings [30,31]. These studies also identified a number of barriers to routinely addressing a patient’s smoking behaviour including time constraints and a perceived need for further training in this area [31].

A sub-analysis of ophthalmologist responses suggests that this profession may be more likely to take a smoking history and encourage patients to quit. The percentage of respondents who reported that they regularly checked on smoking status in the current survey (70%) was considerably higher than previously reported in a larger sample of UK ophthalmologists [32], which could reflect a either a change in awareness over time or response bias.

## Conclusions

There are a number of inherent design limitations of the current study. The overwhelming majority of respondents were optometrists and consequently the reported practices predominantly reflect that of optometrists. Furthermore, the low response rate increases the potential for selection bias, which may have impacted on the reported practices and views expressed. Despite these limitations, the results suggest that many UK eye care professionals are actively engaged in providing nutritional advice to patients with or at risk of AMD.

Although supplements are commonly recommended for those at greater risk of progression to advanced disease, the results of the present study indicated that the majority of supplement recommendations do not comply with current available evidence. Furthermore, since smoking has been identified as a consistent risk factor for the development and progression of AMD, the results also suggest that the assessment of smoking status and the provision of targeted support to quit could be substantially improved. A Cochrane review [33] has confirmed that a brief smoking cessation intervention consisting of simple advice by physicians increases the likelihood that a smoker will successfully quit. By combining such advice with awareness-raising of the link between smoking and AMD, eye care professionals could potentially provide an additional stimulus for smokers to quit. The results of the present study highlight the need for profession-specific guidance to support lifestyle interventions for AMD.

## Abbreviations

AMD: Age-related macular degeneration; AREDS: Age-related Eye Disease Study; CE: Continuing education; GRADE: Grading of Recommendations Assessment, Development and Evaluation; NICE: National Institute for Health and Clinical Excellence.

## Competing interests

The authors declared that they have no competing interests.

## Authors’ contributions

JGL: conception and design of the study, design of the survey questionnaire, analysis of the results and writing the text of the manuscript. JRE: conception and design of the study and design of the survey questionnaire. Both authors read and approved the final manuscript.

## Pre-publication history

The pre-publication history for this paper can be accessed here:

http://www.biomedcentral.com/1471-2458/13/564/prepub
